# Patient and public involvement in preclinical and medical research: Evaluation of an established programme in a Discovery‐Based Medical Research Institute

**DOI:** 10.1111/hex.13968

**Published:** 2024-01-19

**Authors:** Robyn A. Smith, Judith Slocombe, Jo Cockwill, Kathy Minas, George Kiossoglou, Katya Gray, William Lawrence, Michelle Iddles, Clare Scott, Lorraine A. O'Reilly

**Affiliations:** ^1^ Consumer and Community Involvement Theme, Melbourne Academic Centre for Health University of Melbourne Melbourne Victoria Australia; ^2^ Clinical Translation Centre, Cancer Biology and Stem Cells Division and Inflammation Division The Walter and Eliza Hall Institute of Medical Research Melbourne Victoria Australia; ^3^ Department of Medical Biology University of Melbourne Melbourne Victoria Australia

**Keywords:** consumer, evaluation, medical research, patient and public involvement, patient engagement, preclinical research

## Abstract

**Background and Context:**

Involving people with lived experience of health conditions and the public (consumers) in health research is supported by policy, practice and research funding schemes. However, consumer involvement programmes in discovery‐based preclinical research settings are uncommon. Few formal evaluations of these programmes are reported in the literature.

**Objective:**

This study aimed to evaluate an established patient and public involvement programme operating in a major Australian Discovery‐Based Medical Research Institute (DBMRI) to inform programme development and the wider field.

**Design and Participants:**

A multimethods programme evaluation incorporating demographic, descriptive and qualitative data obtained through consumer/researcher co‐developed online surveys and semistructured virtual interviews. Programme participants (*n* = 111) were invited to complete an online survey seeking feedback on their experience of involvement, programme processes and perceived impacts. A purposive sample of 25 participants was interviewed. Descriptive data were analysed using explanatory statistics and qualitative data from surveys and interviews were thematically analysed.

**Results:**

This consumer involvement programme was found to be useful and meaningful for most participants, with specific examples of perceived added value. Consumers most commonly engaged with researchers to inform research development, prepare funding applications or strengthen lay communication of science. Genuine consumer–researcher interactions, relationship development and mutual respect were key elements in a positive experience for participants. Opportunities to ‘give back’, to learn and to ground research in lived experience were identified programme strengths and benefits. Developing researcher training in how to work with consumers, increasing the diversity of the consumer group membership and expanding the range of consumer activities were identified opportunities for improvement. Organisational support and adequate programme resourcing were identified as key enablers.

**Conclusion:**

Discovery‐based preclinical research is often viewed as being distant from clinical application; therefore, consumer involvement may be considered less relevant. However this study identified value in bringing a strong consumer voice to the discovery‐based research process through a coordinated, organisation‐wide approach with the potential for application in similar preclinical research settings.

**Patient or Public Contribution:**

Four consumer partners from the DBMRI Consumer Advisory Panel were actively engaged in developing this programme evaluation. Specifically, these consumer partners co‐developed and pilot‐tested surveys and interview guides, reviewed and commented on project data analysis and reporting and also contributed as co‐authors by editing the manuscript.

## 1 INTRODUCTION

The involvement of patients and the public in health and medical research activities and programmes is an evolving field.^1^, ^2^ Internationally, the term ‘patient and public involvement’ (PPI) is commonly used and reports mostly refer to PPI in individual clinical or health research projects. In Australia, the more common term is ‘Consumer and Community Involvement’ (CCI) and this is considered synonymous with PPI.^3^ We refer to patients, the public, carers/families and service users as ‘consumers’ throughout as it is the term adopted by the study programme participants.

The main public medical research funding body in Australia, the National Health and Medical Research Council, has a policy framework to support consumer involvement^4^ and recently stated, ‘….it is now widely accepted that consumers and community members add value to health and medical research and have a right and responsibility to do so’.^5^ Advocacy groups, such as the Consumers Health Forum of Australia (CHF), have also emphasised the importance of authentic CCI in medical research. The Australia Health Research Alliance (AHRA) (representing >94% of the Australian health and medical research community) and the CHF recently released a Position Statement on Consumer and Community Involvement in health research, which describes the guiding principles for CCI.^6^ Consumer contributions to project development and project review are routinely part of grant application and reporting processes. However, the policy and funding context for medical research organisations has shifted, such that early and ongoing involvement of consumers and community stakeholders is now both expected and strategically important.

While reporting on consumer involvement in research is growing,^1^, ^7^ reports of consumer involvement in preclinical laboratory‐based, discovery or basic science research settings are few.^8^, ^9^ Fox et al.^9^ identified 29 studies in their co‐conducted scoping review of consumer involvement in preclinical research. They did not include any Australian‐based studies and the authors noted that most reports were written from the perspective of researchers rather than consumers. In their review focused on preclinical research consumer involvement, Carroll et al.^8^ documented nine international studies meeting their inclusion criteria. Three of these overlapped with the earlier review. However, again, no Australian studies were included, and only two studies included formal evaluation. While informative, these studies describe engagement in individual projects, advisory groups, workshops or education, mostly related to specific health conditions or a single area of research focus.^8^, ^9^ To date, there is a lack of detailed evaluation of the experience of consumers and researchers in the preclinical research setting, with scant reporting on formal comprehensive programmes operating across entire organisations.^3^, ^8^, ^9^, ^10^, ^11^, ^12^, ^13^, ^14^

This study uniquely focuses on evaluating an established consumer involvement programme available across a leading, Discovery‐Based Medical Research Institute (DBMRI) in Australia (Box 1). The programme under study was one of the first dedicated, resourced consumer‐involvement programmes to commence at a DBMRI in Australia. Volunteer consumers are matched with researchers, with the programme supporting the development of ongoing partnerships to enhance research processes and outcomes. Consumers share their lived experience of health conditions, while researchers share their research ideas, projects and proposals and explore strategies for enhancing science communication. This programme evaluation had three key aims: to explore the programme experiences of consumers and researchers; to identify programme strengths and opportunities for improvement and to inform programme development and the wider field.

BOX 1 **About the Discovery‐Based Medical Research Institute Consumer Program (DBMRICP)**

The DBMRICP has evolved and grown in response to increasing demand since 2013 and is now a structured and resourced programme, available to researchers across the organisation. A Consumer Advisory Panel (CAP) comprising consumers and senior researchers governs the programme and operates under CAP Terms of Reference and a Strategic Plan, endorsed by the DBMRI Board. An annual report on programme activities is prepared for the organisation's Executive and Board. The DBMRICP has a presence on the organisation's website and intranet site and is routinely acknowledged in the Annual Report and Annual General Meeting.

The programme has a dedicated, funded full‐time coordinator and three consumer volunteers who provide significant leadership and operational support. New consumers are recruited into the programme through a combined written expression of interest and interview process and are provided with a formal induction, training programme, access to peer mentoring and a calendar of learning and development activities. Consumers volunteer their time, with reimbursement of out‐of‐pocket expenses (e.g., travel, carparking, refreshments).

Participation is open to researchers in all laboratories, across all five organisational research theme areas. The DBMRICP team offers an annual presentation about the programme in the Institute seminar programme. Researchers request to join the programme through the Program Coordinator and are matched with research area‐relevant consumers. New researchers receive an individual programme introduction from the coordinator. There is no formal researcher training or development programme. The coordinator facilitates the first meeting between researcher(s) and consumer(s), with additional involvement to foster collaboration as required.

Consumers and researchers are predominantly engaged with research funding application development and preparation, with the coordinator's support. One of the key aspects of this programme is that it seeks to establish and build long‐term, ongoing relationships between individual consumers and researchers/laboratory teams. The programme also facilitates connections between consumers (within the consumer cohort) and engagement with the organisation overall.

## 2 MATERIALS AND METHODS

### 2.1 Project context and steering committee

This study was conducted during 2021 when the COVID‐19 global pandemic influenced both the operation of the programme under study and the evaluation. All programme and project activities were conducted virtually. A Steering Committee established to guide the project included consumer and researcher representatives from the DBMRI's Consumer Advisory Panel (CAP), the project sponsor, Program Coordinator and members of the AHRA with expertise in CCI. The evaluation lead (R. A. S.), an experienced health programme evaluator and clinician‐researcher, was employed by the project sponsor and had no pre‐existing relationship with the DBMRI or the programme.

### 2.2 Programme evaluation design

To provide project context and make tacit programme knowledge explicit, the evaluator worked with the project Steering Committee to co‐develop a Program Logic model^15^ for the existing programme.^16^ The multimethods programme evaluation incorporated demographic, descriptive and qualitative data, obtained through co‐developed online surveys, semistructured virtual interviews, content review of programme documentation, support materials and contextual information.

There are multiple existing tools and frameworks for collecting data concerning consumer involvement in medical research.^1^, ^17^, ^18^ However, most are designed for clinical research or health service planning and therefore were of limited use in this preclinical research setting.^8^, ^9^, ^19^ These existing tools predominantly focus on the evaluation of specific projects rather than organisation‐wide programmes that foster involvement in durable research programmes. We considered a range of tools for this study^13^, ^18^, ^20^, ^21^, ^22^ as well as the recommendations of key studies^1^, ^2^, ^7^, ^17^, ^23^, ^24^, ^25^, ^26^, ^27^, ^28^ and an online evaluation tool.^29^ However, we found that there were no ‘off the shelf’ tools suitable for this evaluation.^1^, ^17^, ^23^, ^24^

### 2.3 Survey and interview guide development

Surveys were used to maximise evaluation reach, with subsequent interviews offering greater depth and exploration of experiences. Informed by the literature, the Steering Committee and evaluation lead co‐designed the online surveys and the interview guides (Figures [Link], [Link], [Link]). Surveys included demographic questions, programme experience statements using Likert scale responses and open‐ended free text questions without word limit. The Qualtrics® software platform was used for the online survey data collection and basic summary reports. Interview topic guides aligned with project objectives and were also informed by preliminary analysis of survey data. They explored programme experiences, perceptions of the purpose, impacts and outcomes of consumer involvement in medical research and sought suggestions for programme improvement. Surveys and semistructured interview guides were piloted with CAP consumer and researcher representatives and modified based on feedback.

### 2.4 Survey distribution

All 56 consumers and 55 researchers involved in the programme (as at March 2021) were emailed a survey invitation and hyperlink (excluding researchers and consumers directly involved in the CAP/programme leadership). Two email reminders were sent, with surveys open for 3 weeks.

### 2.5 Semistructured interview and recruitment

Using a matrix of research theme, length of time in the programme, researcher experience, demographic factors and varying interaction styles (e.g., 1:1, 1:team, team:team), a purposive sample of current consumers and researchers was approached for interview. All consumers (*n* = 9) and researchers (*n* = 6) who had exited the programme within the past 2 years, organisational leaders of the five research theme areas of the DBMRI, the senior executive (director) and research office were also invited for interview. Interviews were conducted virtually via Zoom by the evaluation lead, recorded and transcribed verbatim using autotranscription (Microsoft Office 365). A research team member (W. L.) assessed verbatim transcripts against original recordings and corrected errors. Interviewees were offered the opportunity to review the final transcripts; however, no one did.

### 2.6 Data analysis

Descriptive survey data were summarised into tables and graphs. Qualitative data were analysed inductively, reflecting a reflexive thematic analysis approach.^30^, ^31^ The medical research institute essentially operates within a positivist paradigm; however, this evaluation sought to explore participants' reflections on their programme experiences, supporting the value of a reflexive, semantic approach to the qualitative data. Analyses were iterative, involving independent data exploration and review by two researchers (R. A. S. and W. L.), and ongoing discussion between the two to generate themes. Qualitative survey data were first analysed per question, then across questions. Analyses were further developed iteratively, progressively incorporating interview data to build and refine themes. Developed themes were discussed with the Project Steering Group and further refined during the report writing process.

## 3 RESULTS

### 3.1 Study participation

The online survey for consumers (Figure S1) or researchers (Figure S2) was completed by 80% (45/56) and 45% (25/55) of the participants, respectively. Eleven consumers participated in semistructured interviews, including those currently involved in the programme (*n* = 6) and those who had left (*n* = 5 of 9). Eleven researchers participated in semistructured interviews, including those currently involved in the programme (*n* = 9) and those who had left (*n* = 2 of 6). An additional three interviews captured the experiences and views of scientific/organisational leaders. Consumer interviews took an average of 58 min (range 41–90, women *n* = 7). Researcher/leader interviews took an average of 43 min (range 22–62, women *n* = 5).

### 3.2 Survey participant characteristics

**Figure 1:**
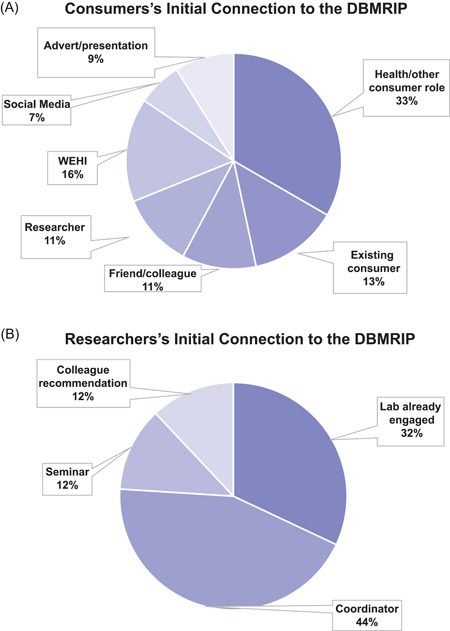
Initial connection with the DBMRICP. Pie chart representation of consumer (A) and researcher (B) means of initially engaging with the DBMRICP programme. DBMRICP, Discovery‐Based Medical Research Institute Consumer Program; WEHI, Walter and Eliza Hall Institute of Medical Research.

Demographic data were obtained from survey respondents. Thirty‐four (75%) consumers identified as female, with 54% aged 60 years or older (Table 1). The majority of consumers had experienced a health condition (*n* = 45, 80%), either directly or as a carer. The consumer group identified as predominantly Anglo‐Celtic (63%). No consumer identified as Aboriginal or Torres Strait Islander. Consumers' initial connection to the programme was varied, with one‐third having consumer advocate roles with other organisations (Figure 1A). The consumer group was highly educated with all except two having completed high school, and the majority (78%) having a tertiary qualification of a Bachelor's degree or above (Table 1). Nineteen consumers (42%) had prior formal training in science, medicine or health care relevant to their area of involvement with the programme. Almost half the consumers (49%) were working in at least part‐time paid employment and based on postcode data, consumers came predominantly from suburbs with an average personal income up to 27% higher than the state average (Australian Bureau of Statistics, accessed 26/02/23).^32^

**Table 1:** Demographics of survey respondents (consumers *n* = 45, researchers *n* = 25).

	Consumers; number (%)	Researchers; number (%)
Gender	Female 34 (75)	Female 16 (64)
	Male 11 (25)	Male 7 (28)
		Prefer not to say 2 (8)
Age range, years		
<40	4 (9)	9 (37)
40–59	12 (27)	11 (45)
60+	N/A	4 (18)
60–69	21 (46)	N/A
70+	8 (18)	N/A
Born in Australia?		
Yes	34 (76)	11 (44)
No	10 (22)	13 (52)
Prefer not to say	1 (2)	1 (4)
Ethnicity/ancestry^a^		
Anglo‐Celtic	32 (63)	12 (48)
European	12 (23)	8 (32)
Asian/other/prefer not to say	7 (14)	5 (20)
Education level		
High school	7 (15)	N/A
Undergraduate	11 (25)	N/A
Postgraduate	24 (53)	N/A
Other	3 (7)	N/A
<10 years PhD		9 (36)
10–20 years PhD		9 (36)
20+ years PhD		7 (28)
Months in the programme		
Up to 12	9 (20)	6 (24)
13–24	16 (36)	6 (24)
25–36	6 (13)	2 (8)
37–48	5 (11)	5 (20)
>49	9 (20)	6 (24)

Abbreviation: N/A, not applicable.

^a^ More than one region could be selected.

Most researcher survey respondents (82%) were aged under 60 years, with just over half born overseas (52%), and most (whether born in Australia or overseas) from an Anglo‐Celtic background (48%). A variety of programme entry points were available for researchers (Figure 1B) and all were PhD qualified, had a range of experience levels (emerging, established and senior) and varying programme participation timeframes (Table 1). Researchers from all five organisational research themes were represented within the programme, with 43 of 83 research laboratories (52%) having active consumer input. Fewer than 20% of the organisation's 380 researchers were registered as programme participants at the time of study recruitment (March 2021).

### 3.3 What do consumers do?

Consumer and researcher survey respondents offered their views on the purpose and role of consumers in health and medical research (Table 2). Consumer and researcher survey respondents indicated how frequently they participated in a range of consumer involvement activities (Table 3). Research grant application assistance was the most frequent contribution that consumers made within this programme (Table 3). Giving feedback on presentations and sharing research results was also a frequent activity (Table 3). Partnership record‐ keeping is an activity that consumers are expected to complete as part of the programme, with 80% indicating that they do this often or sometimes (Table 3). Researchers and consumers differentially rated the frequency of consumers providing mentoring or personal support to researchers/teams (Table 3). Discussing future research, research questions and research planning was identified as often or sometimes occurring by 49% of consumers and 64% of researchers (Table 3). Most respondents in both groups indicated that assisting with manuscript preparation, fundraising support and networking support were activities that the programme consumers rarely or never engaged in.

**Table 2:** Summary of survey respondents' views on purpose and role of consumers in health and medical research (consumers *n* = 45, researchers *n* = 25).

Consumer responses	Researcher responses
To ensure that the voice of those impacted by the research is heard and that they/we are involved in planning/developing/contributing to the research	Offering patient/carer/survivor/community perspectives to ground the research in the ‘real world’
To ensure that the lived experience of people with health conditions/carers is heard and incorporated into the research	Helping to focus the researcher and the research questions to areas with impact and potential benefit for consumers/patients/end users—a ‘reality check’
To bring a public/lay perspective to the science	Improving the communication of science in lay terms and helping scientists make their work easier to understand
To strengthen the accountability of scientists/health and medical researchers	Sharing two‐way communication about the science (between scientists and consumers/lay audience/public)
To make science/health and medical research more visible in the community—providing a bridge between the scientists and the public/community	To provide a community link that helps to promote health and medical research and the MRI; offering an advocacy role
To help with targeting research efforts by asking questions and exploring the practical application or translation of science	To support research grant writing and the development of fundable proposals
To assist scientists to keep their work in perspective and to foster a broader, consumer informed perspective	Reminding scientists of the importance of their work and the expected outcomes of their work
To ensure that the language used and communication about the science are easy to share and understand	

Abbreviation: MRI, Medical Research Institute.

**Table 3:** Survey responses to statements about what consumers do (researchers *n* = 25, consumers *n* = 45).

Activity	Respondent	Often		Sometimes		Rarely		Never	
		*n*	%	*n*	%	*n*	%	*n*	%
Grant application assistance	Consumer	22	49	14	31	5	11	4	9
	Researcher	15	60	7	28	3	12	0	0
Giving feedback to the researcher or me/my team about presentations and sharing research results	Consumer	10	22	15	33	7	16	13	29
	Researcher	4	16	13	52	5	20	3	12
Diary/record keeping regarding the partnership	Consumer	21	47	15	33	5	11	4	9
	Researcher	5	20	2	8	10	40	8	32
Mentoring the researcher/team or me/my team	Consumer	2	4	8	18	13	29	22	49
	Researcher	5	20	7	28	7	28	6	24
Talking about MRI research as a consumer representative (e.g., to community organisations, conferences)	Consumer	0	0	6	13	12	27	27	60
	Researcher	2	8	10	40	7	28	6	24
Involved in writing papers and publications regarding the research	Consumer	0	0	3	7	5	11	37	82
	Researcher	0	0	7	28	7	28	11	44
Teamwork/team‐building support	Consumer	1	2	8	18	11	24	25	56
	Researcher	2	8	9	36	4	16	10	40
Fundraising support for researcher/team or my research/team	Consumer	2	4	1	2	4	9	38	85
	Researcher	0	0	5	20	7	28	13	52
Networking/connection building	Consumer	3	7	7	16	9	20	26	57
	Researcher	0	0	7	28	11	44	7	28
Personal support to the researcher/team or me/my team	Consumer	2	4	16	36	10	22	17	38
	Researcher	5	20	13	52	4	16	3	12
Discussing future research, research questions or research planning	Consumer	4	9	9	40	9	20	14	31
	Researcher	7	28	1	36	6	24	3	12
Other role: If yes, please describe and select how often. If no, please select ‘never’	Consumer	4	9	1	2	0	0	40	89
	Researcher	2	8	2	8	0	0	21	84

Abbreviation: MRI, Medical Research Institute.

### 3.4 What is the experience like of being a consumer at this DBMRI?

The majority of consumers indicated that their opinion is listened to (74%) and respected (74%) and that they were comfortable working with their researcher (65%) (Table 4). Thirty‐six consumers (80%) agreed with the statement that they were a *valued* member of the research team and 51% agreed that they were an *integral* part of the research team. The majority of consumers (69%) agreed that they sometimes struggled to understand the science or technicalities of the research, and 51% indicated that they had sufficient opportunity to contribute to the research (Table 4).

**Table 4:** Survey responses—what is the experience like of being a consumer at this DBMRI? (consumers *n* = 45).

Statement	Strongly agree		Somewhat agree		Neither agree nor disagree		Somewhat disagree		Strongly disagree	
	*n*	%	*n*	%	*n*	%	*n*	%	*n*	%
My opinion is listened to	33	74	11	24	1	2	0	0	0	0
My opinion is respected	33	74	11	24	1	2	0	0	0	0
I sometimes struggle to understand the science and technicalities of the research that I work on	9	20	22	49	2	4	9	20	3	7
I am an integral part of the research team/programme	9	20	14	31	13	29	5	11	4	9
I have enough opportunities to contribute to the research	13	29	10	22	15	34	5	11	2	4
I feel comfortable working with my researcher(s)	29	65	14	31	2	4	0	0	0	0
In my experience, consumer involvement in research at the DBMRI is more of a ‘tick box’ exercise	3	7	6	13	9	20	8	18	19	42
I am a valued member of the research team	19	42	17	38	5	11	3	7	1	2
I feel that the expectations are too high on me as a consumer	0	0	1	2	10	22	17	38	17	38
The researcher(s) I work with sometimes struggle to understand my perspective	0	0	1	2	5	11	15	33	24	54
I sometimes struggle to understand my researcher's perspective	1	2	3	7	8	18	20	44	13	29
I am able to share my experience and knowledge with my researcher(s)	22	49	17	38	5	11	0	0	1	2
In my experience, consumer involvement in research at the DBMRI is really believed in	23	51	14	31	7	16	0	0	1	2

Abbreviation: DBMRI, Discovery Based Medical Research Institute.

Most consumers (87%) agreed that they could share their knowledge and perspective with their researchers and that consumer involvement at the DBMRI ‘is really believed in’ (81%). Twenty‐seven consumers (60%) disagreed that consumer involvement at the DBMRI is a ‘tick box’ exercise (Table 4).

### 3.5 What is the experience like of being a researcher, working with consumers in this programme?

Researchers indicated that they respected (64% strongly agree) and listened to their consumers' opinions (56% strongly agree) (Table 5). Consumers were considered valued team members (48% strongly agree) and the majority of researchers were comfortable working with their consumers (60% strongly agree) (Table 5). A small proportion (20%) of researchers agreed or strongly agreed that they sometimes struggled to explain the science and technicalities of the research to their consumer (Table 5). Overall, researchers indicated that they felt able to share their experience and knowledge with their consumer (96% agreed or strongly agreed), with few (8%) agreeing that they sometimes struggled to understand the consumer perspective. The majority of researchers (96%) had enough opportunity to engage with their consumer(s) about research and agreed that consumer involvement with research at the DBMRI was ‘really believed in’ (76%).

**Table 5:** Survey responses—what is the experience like of being a researcher engaged with consumers at the DBMRI (researchers *n* = 25).

Statement	Strongly agree		Agree		Neither agree nor disagree		Disagree		Strongly disagree	
	*n*	%	*n*	%	*n*	%	*n*	%	*n*	%
I have enough opportunities to engage with my consumer(s) about research	10	40	14	56	1	4	0	0	0	0
I listen to my consumers' opinion	14	56	9	36	2	8	0	0	0	0
I sometimes struggle to explain the science and technicalities of my research to my consumer(s)	1	4	4	16	3	12	13	52	4	16
I respect my consumers' opinion	16	64	8	32	1	4	0	0	0	0
Consumers are a valued member of the team	12	48	9	36	3	12	1	4	0	0
I feel comfortable working with my consumer (s)	15	60	9	36	1	4	0	0	0	0
In my experience, consumer involvement in research at the DBMRI is more of a ‘tick box’ exercise	1	4	4	16	3	12	14	56	3	12
The consumer(s) I work with sometimes struggle to understand my perspective	0	0	5	20	7	28	9	36	4	16
I am able to share my experience and knowledge with my consumer(s)	7	28	17	68	1	4	0	0	0	0
I sometimes struggle to understand my consumers' perspective	0	0	2	8	3	12	14	56	6	24
I feel that the expectations are too high on me as a researcher working with a Consumer	0	0	1	4	6	24	12	48	6	24
In my experience, consumer involvement in research at the DBMRI is really believed in	4	16	15	60	4	16	2	8	0	0

Abbreviation: DBMRI, Discovery Based Medical Research Institute.

### 3.6 Reasons for involving consumers—accountability, meaning and contribution

Many and multiple reasons for involvement were described in surveys and interviews, in medical research broadly and as part of the programme specifically. Across all participant groups, considering research through a consumer lens was seen as a public accountability mechanism and obligation, informing research focus and priorities, and assisting in identifying what is important. Grounding the research in lived experience was identified as a particular need within a DBMRI, since many researchers within this setting have nonclinical backgrounds and would not otherwise have access to consumer voices. ‘[The purpose of involvement is….] To challenge researchers by keeping consumer issues – what matters to the patients and families who will be the ultimate beneficiaries of their research – front and centre. It brings a different perspective to the table’ (Consumer Interview).

Consumers described involvement in research as a way to find meaning from a significant health experience, and an expressed desire to ‘give back’ by contributing to research that seeks a cure or to improve future treatment options for others. Sharing the lived experience of a health condition, making a meaningful contribution to science and improving the visibility of science by building a bridge between scientists and the community were also given as reasons for involvement in preclinical research. Many consumers identified opportunities to contribute to a researcher's professional and personal growth by supporting communication skill development, providing encouragement and mentoring.

Researchers additionally identified instrumental and personal aspects of engaging with consumers. Improving science communication in lay terms, promoting and advocating for research, meeting grant requirements, developing fundable proposals and reminding scientists of the importance of their work were reasons to engage [Why involve consumers in research…] ‘To allow researchers to reflect on the direction of their research – to always keep in mind the ultimate goal – treatments or cures. To provide feedback on layperson's statements for grants etc. To help hone communication skills. Consumers provide motivation and “reality checks”’ (Researcher Survey). An opportunity to learn was also identified as a reason and driver of participation for both consumers and researchers. For consumers, the opportunities centred around four learning areas: (1) knowledge about their own health condition; (2) science and the research process; (3) the organisation and (4) the life of career researchers. For researchers, learning centred on acquiring knowledge about the specific health condition that they were researching and the real‐life experiences of consumers.

### 3.7 Adding value to research

The surveys asked whether consumer involvement adds value to the DBMRI's research. Thirty‐eight consumers (84%) agreed, 16% selected ‘maybe’ and none disagreed. Researchers agreed (75%) or answered ‘maybe’ (25%) and none disagreed. Open text survey and interviewee responses revealed participants' views on adding value including grounding research in lived experience, bringing a different perspective and relevance to the research, accountability to the wider community, improving science communication and fostering learning and motivation. For a proportion of respondents, adding value had an element of conditionality: that the partnership dynamics needed to be conducive to enable an active contribution. Some researchers also gave examples attributing individual career and grant successes to consumer involvement and identified direct value added when writing grant applications ‘…….[they] really made me think about what the impact on the outcome of my research would be. Rather than just being caught in the nittty gritty of explaining this one particular thing. I would have to think of, like, what the broader implications of that are. So I found it really helpful’ (Researcher Interview). For those less certain about ‘added value’, reservations related to the time investment required by both consumers and researchers to develop meaningful, functional relationships, the challenges of highly technical science and the challenges of enabling meaningful consumer contribution and genuine input [Does involvement add value?] ‘The answer I want to give is not “maybe” but “sometimes.” Sometimes it does and sometimes it does not add value. Some researchers value consumer input and some do not. In as much as evidence of consumer involvement is a requirement of some funding, it adds value in making an application eligible’ (Consumer Survey).

### 3.8 Good relationships are a key to success

Successful programme outcomes were felt to rely on well‐matched consumers and researchers, and on support structures that enabled them to engage and develop genuine relationships founded on mutual trust and respect. When these conditions existed, participants described a range of positive personal impacts from involvement. For both researchers and consumers, these included confidence boosting, a renewed sense of purpose, personal growth and development and increased motivation to contribute and to work collaboratively on the science.

Researchers spoke about receiving consumer support and mentoring that helped them to ‘ride the rollercoaster’ of a science career and learn new skills, particularly in communication. Consumer partners were valued as trusted mentors and friends in some of the well‐established relationships. Many consumers derived a sense of belonging, a sense of altruism and described making a contribution to a worthwhile endeavour, having an opportunity to ‘give back’ and having an opportunity to learn through partnering with researchers. Researcher attitude was identified as a key factor for developing and sustaining collaborative researcher/consumer partnerships. It was identified as a significant barrier when consumers felt that engagement was not genuine, partnerships were unequal or focused merely on ‘ticking a box’ on a research grant application. It was enabling when researchers were open‐minded, prepared to listen to consumer input to research and learnt from consumers sharing their lived experience of a health condition‘….we had a meeting for an hour where we discussed our grant …. and then we said ‘that's it’ and then we went for a drink. You know, so we, we did some work but we also could just sit back and, and enjoy the relationship too’ (Senior Researcher Interview).

Consumer and researcher interviewees observed that researcher engagement in the programme may be initiated as a ‘tick box’ exercise, driven by granting body requirements, but evolve over time into genuine collaborations, building mutual trust and respect. Many participants also described instrumental factors required to underpin the development of relationships. These included having clear organisational support for consumer involvement, an active and accessible programme coordinator who could facilitate and ‘trouble shoot’ connections and training and ongoing support for both consumers and researchers.

### 3.9 Negative experiences and managing expectations

Approximately a quarter of the participants described limiting factors or a negative experience during their time in the programme. For consumers. these included COVID‐19‐specific challenges that affected relationships or reduced their level of involvement, knowledge gaps and rapport and power imbalance issues between consumer and researcher. Individual examples included not feeling heard by the researcher/research team, feedback was not being taken on board or that some researchers did not communicate effectively. For some of those choosing to leave the programme, communication issues and experiences of unequal or non‐genuine engagement predominated. Many of the negative experiences seemed to relate to underlying mismatched or unclear expectations of the consumer role and the extent of involvement.

Researchers also described mismatched or unclear expectations concerning roles or the requirements of the programme. Individual examples included issues with timing of communication/consumer feedback, particularly late in the grant application process; poor communication when programme processes or expectations seemed to have changed; and a situation where consumers had a strong emotional connection to the health issue that was challenging for the researchers/consumers and programme to navigate. All researchers exiting from the programme in the previous 2 years did so due to an employment change, unrelated to the programme.

### 3.10 COVID‐19 and consumer involvement

Public Health guidance during the COVID‐19 pandemic drove ongoing programme adaptations, particularly given the vulnerable health status of many consumer volunteers. All programme activities moved online. The programme changes due to COVID‐19 generated positive and negative outcomes and participant reactions (Table 6).

**Table 6:** Summary of COVID‐19 impacts on the programme experience drawn from surveys and interviews.

Pros of virtual interactions	Cons of virtual interactions
Additional education and learning opportunities for consumers (e.g., hearing from researchers about current COVID research, joining a wide range of MRI seminars).	Limited opportunities for informal connection and incidental conversation (e.g., meeting over coffee after a formal meeting or lecture).
No travel time or parking issues.	Harder to establish/maintain relationships in some instances.
Formal DBMRICP training easily delivered virtually (e.g., lectures, presentations).	Virtual interactions can feel stilted or static when compared with in‐person interactions.
Consumers able to more easily and regularly attend lab meetings virtually.	Less contact between researcher/consumers in some instances— particularly with changes to the activity of the research institute (e.g., fewer grants applications being written, researchers working from home, other changes to the nature of work).
More contact between some researcher/consumer groups as meetings were easier to arrange and participate in.	Loss of momentum for some relationships and for the programme overall.
Zoom consumer meetings better attended than in‐person sessions (e.g., programme induction).	Increased family and work pressures during COVID‐19 resulting in reduced availability/flexibility for programme participation.

Abbreviation: DBMRICP, Discovery‐Based Medical Research Institute Consumer Program.

## 4 DISCUSSION

As highlighted in recent systematic reviews,^8^, ^9^ few preclinical consumer involvement programmes are reported in the literature and evaluation of involvement is needed.^33^ This study set out to evaluate an established, coordinated, organisation‐wide involvement programme in a preclinical setting, with the aim of informing programme improvement and the wider field. Discovery‐based preclinical research is viewed as not public facing and somewhat distant from clinical application; therefore, involvement of consumers could be perceived to be less relevant.^8^, ^9^, ^19^ Participants in this study held a different view. They confirmed the value in bringing a consumer voice to the discovery‐based research process.

Reflecting key Australian research policy and practice^4^, ^6^ and international literature,^8^, ^9^ researchers, consumers and organisational leaders in this study consistently expressed the belief and expectation that research outcomes should ultimately benefit the community and that those who have experienced health conditions should be genuine stakeholders in the research process, outputs and outcomes of the organisation. These beliefs and accountabilities underpinned the consumer programme.

The importance of fostering genuine consumer involvement, and avoiding tokenism, is a recurring theme in the literature.^23^, ^25^, ^34^, ^35^, ^36^, ^37^ In this DBMRI, there was an overall sense of clear organisational support for the programme and for active consumer involvement in research. This support was fundamental to enabling both researchers and those with lived experience to engage actively in the research process. Within this positive organisational context, some consumers described experiences of ‘tick box’ initial or individual interactions with researchers. The experience of feeling undervalued influenced some consumers' decisions to leave the programme and was cited as a source of disappointment or frustration for those seeking deeper collaborations or co‐design experiences. These findings highlight the importance of acknowledging the significant impact that consumer involvement can have for individuals.^3^ The need to continuously foster genuine engagement and address tokenism are essential elements of any programme that require ‘space to talk’ and ‘space to change’ ^38^ to enable the research co‐production process.

Engaging in the consumer programme was an important experience for both consumers and researchers, with sometimes quite profound reported impacts. Those who experienced the programme as beneficial valued the opportunities to learn from one another, to make a meaningful contribution to the work, the science and the organisation. The feeling of giving back and making a difference was a strong motivator for consumers and researchers. Consumers also valued the opportunity to contribute to advancing science and to play a bridging role between scientists and the community, reflecting previous reports.^8^, ^9^ The style of involvement and range of activities are important considerations in any involvement programme. For instance, while the majority (96%) of researchers in this programme felt that they had sufficient opportunity to engage with consumer(s), only half of the consumers agreed that they had sufficient opportunity to engage. Research priority setting is the primary focus of consumer involvement in most preclinical research reported in the literature.^8^, ^9^ In contrast, we found that consumers and researchers most commonly engaged around the research proposal and grant development process, exploring research questions and developing science communication and lay presentation skills. Research priority setting, publication and dissemination, translation of outcomes and community engagement and philanthropy were less common. Although the organisation describes these areas of activity as being extremely important, contributions in these areas were less frequent (Table 3). In future, there is scope to extend consumer roles to other areas of collaboration.^13^, ^14^

The complex nature of discovery‐based research and the current programme model has prompted the recruitment of a highly skilled consumer cohort. The consumers engaged within this programme typically have higher education levels than the general population,^39^ professional or science relevant backgrounds and significant work and life experience to contribute, in addition to their lived experience of a health condition. At least 30% of consumers were experienced advocates, with research involvement roles in other organisations. Similar cohort characteristics, particularly in basic sciences research, have been reported,^33^ highlighting the challenge for consumers to be ‘research aware’ with specific specialist knowledge in order to engage with this type of research. We found that researchers and their consumer partners form working relationships centred on research, with some also developing mentoring arrangements or friendships that extended beyond the realm of the science/programme. This appears to produce positive benefits, but also poses a potential challenge. First, consumer/researcher teams in the programme who do not develop this level of relationship can feel a sense of being ‘lesser’ by comparison. Second, if consumers become so embedded in the team (‘insiders’), are they still able to bring the ‘outsider’ perspectives that can be so useful in the research process.?^28^ This is an ongoing tension for the programme.

This may be addressed to some extent through approaches to improve the diversity of participation. In addition to high educational levels and professional or scientific backgrounds, the majority of the consumer cohort resided in higher‐than‐average earnings postcodes and were from predominantly Anglo‐Celtic backgrounds. This cohort is not representative of Australia's diverse population and the lack of diversity in consumer programmes is an on‐going issue in medical research^9^, ^34^, ^40^ and something requiring specific strategies to address^8^, ^9^, ^40^ The programme has begun to tackle this challenge by more targeted recruitment and by developing larger consumer ‘teams’ comprised of consumers with more diverse backgrounds and with complementary skills to work together as a group to engage with researchers.

Feedback from researchers, consumers and leaders highlighted the importance of setting clear expectations from the outset of involvement in the programme. The CHF/AHRA Position Paper^6^ recommends that both researchers and consumers receive comprehensive orientation and ongoing training and support to ensure that they are equipped with the skills to enable relationships to develop and work smoothly. Formal training is considered fundamental to the consumer involvement process^41^ and to addressing potential power imbalances between researchers and consumers.^34^ At the time of the study, a formal induction, training and mentoring programme existed and was made available for consumers, but not for researchers. This discrepancy had previously been identified, but had not yet been addressed by the programme team. Better preparing researchers to work with consumers was identified by consumers, researchers and leaders in this study as an important priority and a key factor in better managing expectations and realising the full potential of consumer/researcher partnerships. Training is not the only strategy to improve programme deficits; careful matching of researchers with consumers was strongly felt to be a key success factor in this type of programme. Having a skilled coordinator with dedicated time to engage participants, facilitate effective relationships, manage expectations, provide ongoing support and ‘trouble shoot’ emerging issues was also identified as a key success factor. These findings add weight to the existing literature describing ‘engagement support’ and facilitation as essential elements in preclinical programmes.^3^, ^14^, ^19^

The COVID‐19 pandemic undoubtably impacted the programme. The negative impacts of lockdowns and meeting restrictions were identified by some consumers and researchers as particularly affecting relationships, engagement and momentum (Table 6). For many others, the flexibility of virtual participation was either a neutral or a positive experience. Overall, the COVID‐19 pandemic has resulted in positive changes for the programme. The experience of virtual interaction has consequently further expanded opportunities for online or blended programme activities that might otherwise have been difficult to implement.

While there have been reports of consumer participation in research, these have not generally explored programme limitations or negative experiences.^7^ A strength of our approach was to specifically invite feedback on negative experiences and programme limitations in order to better understand programme challenges and development opportunities. We also approached those who had left the programme within the past 2 years to understand their experiences and reasons for departure. It was challenging to conduct this study during the COVID‐19 pandemic, when additional stressors and novel working modes affected participants, the programme's operations and evaluation. Conducting the evaluation virtually did enable inclusion of interview participants from any geographic location; however, it possibly also reduced the capture of some of the subtleties and nuance of face‐to‐face engagement. To minimise this risk, an experienced researcher conducted interviews at a time convenient to interviewees.

Adapting to the constantly changing external environment may have affected study participation. The consumer response rates were high (>80%) and the researcher response rate was lower (45% for surveys, ~50% combining surveys and interviews). To give perspective, this is a higher response when compared with other in‐house staff surveys conducted in this organisation (typically ∼30%–35%). It remains conceivable that we have captured the experiences and views of those more engaged in the programme.

At the time of the study, 43 of 83 laboratories (52%) had consumers involved and fewer than 20% of the organisations' researchers were formally and voluntarily registered in the programme. The programme team report that the researcher involvement rate is likely higher than 20% as consumers may attend a laboratory meeting with multiple researchers, although only one researcher from the group is formally registered with the programme. As we drew respondents only from those formally registered, we may have omitted additional researchers who had engaged with consumers outside the formal programme.

## 5 CONCLUSIONS

This study contributes to the currently limited knowledge base relating to the involvement of consumers, community members and the public in research being conducted in preclinical, discovery‐based settings. The evaluation demonstrated that a well‐supported programme that aimed to build long‐term relationships between researchers and consumers in a preclinical setting was both useful and meaningful for the majority of participants. Establishing and supporting effective consumer/researcher relationships through a resourced, facilitated consumer involvement programme delivers a range of benefits to those involved. This programme offers a potential model for involving consumer partners in preclinical research, with lessons learned about improving programme activities and experiences, and broadening consumer diversity and contribution.

## AUTHOR CONTRIBUTIONS

**Robyn A. Smith**: Conceptualisation; investigation; formal analysis; writing—original draft; methodology; validation; data curation; writing—review and editing; software; supervision; resources. **Judith Slocombe**: Methodology; writing—review and editing. **Jo Cockwill**: Methodology; writing—review and editing. **Kathy Minas**: Methodology; writing—review and editing. **George Kiossoglou**: Writing—review and editing; methodology. **Katya Gray**: Writing—review and editing; project administration; resources. **William Lawrence**: Validation; data curation; formal analysis; project administration. **Michelle Iddles**: Funding acquisition; conceptualisation. **Clare Scott**: Conceptualisation; writing—review and editing; project administration; supervision. **Lorraine A. O'Reilly**: Conceptualisation; supervision; writing—original draft; writing—review and editing; project administration; methodology.

## CONFLICT OF INTEREST STATEMENT

Katya Gray is the Program Coordinator of the Discovery‐Based Medical Research Institute Consumer Program. The remaining authors declare no conflict of interest.

## ETHICS STATEMENT

Ethical approval was granted by the Walter and Eliza Hall Institute Human Research and Ethics Committee (Project #20/22, December 2020).

## Supporting information


**Figurementary Figure 1**. Online survey for consumers.

**Figurementary Figure 2**. Online survey for research.

**Figurementary Figure 3**. Interview Guide.

## Data Availability

The survey data reported in this article are not publicly available to meet the terms of ethical approval for the study and to protect participant privacy.
