# Supplementary material for: Patient and public involvement in preclinical and medical research: Evaluation of an established programme in a Discovery‐Based Medical Research Institute
Source: Health Expect. 2024 Jan 19;27(1):e13968. doi: 10.1111/hex.13968 (PMC10797251; doi:10.1111/hex.13968)
Supplement: Supplementary file 1 — Figurementary Figure 1. Online survey for consumers. [file HEX-27-e13968-s003.docx]

Consumer Program Evaluation - Consumer

Q1.1 By clicking the button below, you acknowledge:
Your participation in the study is voluntary. You are 18 years of age. You are aware that you may choose to terminate your participation at any time for any reason.

- I consent, begin the study
- I do not consent, I do not wish to participate

*Becoming a Consumer; please consider the experience of joining the program*

Q2.1 *How did your involvement in the DBMRI Consumer Program come about? (eg: How did you first become aware of the opportunity to become a Consumer with the DBMRI?) Open: no limit.

Q2.2 *Why did you decide to become a Consumer at the DBMRI? Open: no limit.

Q2.3 *In your opinion, what is the purpose and role of consumer involvement in research? Open: no limit.

Q2.4 *In which DBMIR Theme area do you mainly contribute as a Consumer?

Multiple choice – Select one of 5 theme areas or Not sure

Q2.5 *What was it like meeting your researcher/team for the first time? How did you feel? Open: no limit.

Q2.6 *How well prepared did you feel for engaging with a researcher when you first started working together where 1 is *Not at all prepared* and 10 is *Ideally prepared*.

Select from 1 to 10.

Q2.7 Please comment on your rating.........(eg: what does or could contribute to being ideally prepared?) Open: no limit.

Q2.8/2.9 *Please reflect on your experiences of the DBMIR Consumer Program and respond to each of the following statements.

|  | Strongly agree | Agree | Neither agree nor disagree | Disagree | Strongly disagree | Not Applicable |
| --- | --- | --- | --- | --- | --- | --- |
| The initial induction and orientation made my role clear |  |  |  |  |  |  |
| The initial training prepared me for taking up my role as a consumer |  |  |  |  |  |  |
| I understand what is expected of me as a consumer |  |  |  |  |  |  |
| I am aware of the Consumer training options available through the DBMRI |  |  |  |  |  |  |
| I can easily access the Consumer Training options available through the DBMRI |  |  |  |  |  |  |
| I can access adequate support through DBMRI for my Consumer role |  |  |  |  |  |  |

| The workshops and training available through DBMRI Program help me in my role as a Consumer |  |  |  |  |  |  |
| --- | --- | --- | --- | --- | --- | --- |
| I have a direct say in when I meet/work with my researcher/s |  |  |  |  |  |  |
| The times that we meet/work together are convenient for me |  |  |  |  |  |  |
| I can easily access the Coordinator of the DBMRI Consumer Program |  |  |  |  |  |  |
| I feel my researcher(s) was (were) well prepared for working with me |  |  |  |  |  |  |
| Working with my researcher(s) takes up too much of my time |  |  |  |  |  |  |

Q2.10 Please add any comments or feedback about your experiences of the coordination and organisation of the Consumer Program..........Open: no limit.

Q2.11 As a Consumer do you have a mentor (from within the DBMRI Consumer program)?

- Yes
- No

Q2.12 If Yes: how useful is the mentoring relationship in supporting you in your Consumer role?

- Extremely useful
- Very useful
- Moderately useful
- Slightly useful
- Not at all useful

Q2.13 If no, how could a mentor support you in your Consumer role? Open: no limit.

*Being a Consumer*

Q3.1 In what ways do you contribute to the work of your researcher/team/lab?

Please select an answer for each way of working, *including* "other role".  Please select "never" for any that do not apply.

|  | Often | Sometimes | Rarely | Never |
| --- | --- | --- | --- | --- |
| Grant application assistance |  |  |  |  |
| Giving feedback to the researcher about presentations and sharing research results |  |  |  |  |
| Diary/record keeping regarding the partnership |  |  |  |  |
| Mentoring the researcher/team |  |  |  |  |
| Talking about DBMRI research as a Consumer representative (eg: to community organisations, conferences) |  |  |  |  |
| Involved in writing papers and publications regarding the research |  |  |  |  |
| Teamwork/team building support |  |  |  |  |
| Fundraising support to the researcher/team |  |  |  |  |
| Networking and connection building |  |  |  |  |
| Personal support to the researcher/team |  |  |  |  |
| Discussing future research, research questions or research planning |  |  |  |  |
| Other role: If yes, please describe & select how often. If no, please select "never" |  |  |  |  |

Q3.2 *Who usually organises activities and meetings with your researcher(s)?

- The DBMRI Consumer Program Coordinator
- I do
- My researcher(s) does(do)
- Other, please describe (Open: no limit).

Q3.3 *How do you usually interact and work with your researcher(s)?  Please consider each way of working, *including* "Other".  For each way of working, please select Yes/No.

|  | Yes - regularly | Yes - occasionally | No |
| --- | --- | --- | --- |
| Meetings (face-to-face) |  |  |  |
| Meetings via Zoom/Virtually |  |  |  |
| Phone calls |  |  |  |
| Emails |  |  |  |
| Sharing documents via electronic platforms (eg: Teams or Dropbox) |  |  |  |
| Meeting with others outside DBMRI regarding the research (eg: community groups, funding bodies, advocacy) |  |  |  |
| Other; If yes, please describe |  |  |  |

Q3.4 *In your experience, which approach or combination of approaches to working together are best and why? Open: no limit.

Q3.5 **Including* ***all*** *the different ways of working together*, please select the option that best reflects approximately how often you are in contact with your researcher(s).

- Multiple times per week
- About weekly
- About fortnightly
- About monthly
- About every 6 - 8 weeks
- About quarterly (approx. 4 times/year)
- About twice a year
- Only when there is a grant application to write. Please state approximately how often this occurs in a year. (Open; no limit)

Q3.6 *What is the experience like of being a Consumer engaged with researchers at the DBMRI? Please respond to all statements.

|  | Strongly agree | Somewhat agree | Neither agree nor disagree | Somewhat disagree | Strongly disagree |
| --- | --- | --- | --- | --- | --- |
| My opinion is listened to |  |  |  |  |  |
| My opinion is respected |  |  |  |  |  |
| I sometimes struggle to understand the science and technicalities of the research I work on |  |  |  |  |  |
| I am an integral part of the research team/program |  |  |  |  |  |
| I have enough opportunities to contribute to the research |  |  |  |  |  |
| I feel comfortable working with my researcher(s) |  |  |  |  |  |
| In my experience, Consumer involvement in research at the DBMRI is more of a "tick box" exercise |  |  |  |  |  |

| I am a valued member of the research team |  |  |  |  |  |
| --- | --- | --- | --- | --- | --- |
| I feel that the expectations are too high on me as a Consumer |  |  |  |  |  |
| The researcher(s) I work with sometimes struggle to understand my perspective |  |  |  |  |  |
| I sometimes struggle to understand my researcher's perspective |  |  |  |  |  |
| I am able to share my experience and knowledge with my researcher(s) |  |  |  |  |  |
| In my experience, Consumer involvement in research at The DBMRI is really believed in |  |  |  |  |  |

Q3.8 *In your opinion, does consumer involvement bring added value to DBMRI research?

- Yes
- Maybe
- No

Q3.9 *Please comment on your answer about the added value of consumer involvement to DBMRI research. (eg: why/why not......what underpins your opinion?) Open: no limit.

Q3.10 *Do you think that the *researcher(s)* you work with share your view about the added value of consumer involvement to DBMRI Research?*

- Definitely yes
- Probably yes
- Might or might not
- Probably not
- Definitely not

Q3.11 *Please comment on your answer............ Open: no limit.

Q3.12 *How do you judge whether consumer involvement has made a difference to a study or research program you are involved in? Open: no limit.

Q3.13 Please give any examples of how an application, project, program or other aspect of DBMRI research has been changed because of consumer involvement..........Open: no limit.

Q3.14 Please describe anything that has come from the partnership between you and the researcher(s) that you were not expecting or that has surprised you? Open: no limit.

Q3.15 *What do you think are the downsides of consumer involvement in research? Open: no limit.

Q3.16 *Has anything gone wrong or had a negative impact during your involvement in the Consumer Program at the DBMRI?

- Nothing I can think of
- Yes, please describe Open: no limit.

Q3.17 What, if anything, have you learned from the experience of being a Consumer at the DBMRI? Open: No limit.

Q3.18 *What do you get from the experience of being a Consumer at the DBMRI? Open: No limit.

Q3.19 What more, if anything, could Consumers do to contribute to DBMRI research? Open, No Limit.

*Advice and future planning*

Q4.1 *What do you think are the best ways to enable consumer involvement in research to work well?  What needs to be in place? Open: no limit.

Q4.2 *What do you think are the challenges or barriers to consumer involvement in research working well?  Open: no limit.

Q4.3 *What would you change or improve about the DBMRI Consumer Program? Open: no limit.

Q4.4 *Please describe how COVID-19 has impacted your participation as a Consumer. Open: no limit.

*About You*

Q5.1 *At the start of 2021, approximately how long had you been involved as a consumer at DBMRI? Please answer in months (eg: 1 year = 12; 2.5 years = 30; 4 years = 48)

Q5.2 *What is your age?

- < 20
- 20 - 29
- 30 - 39
- 40 - 49
- 50 - 59
- 60 - 69
- 70 - 79
- 80 +

Q5.3 *What is the highest level of education you have completed or the highest degree you have received?

- High School (up to Year 11)
- High school graduate (HSC/VCE or equivalent)
- VET qualification
- Bachelor's degree
- Post Graduate Diploma
- Master's degree (6)
- Professional degree (JD, MD)
- Doctoral degree/PhD
- Other, please specify (Open)

Q5.4 *Do you have any *formal* training in science, medicine or health care that is relevant to the area(s) you support as a DBMRI Consumer?

- No
- Yes (please describe)

Q5.5 *Which response best describes your primary work situation?

- In paid part-time work/self-employed part-time
- In paid full-time work/self-employed full-time
- Retired from paid work
- Unable to work because of long-term disability or health.
- Primary carer (eg: carer for family/dependents)
- Unemployed
- In full-time education or training (including government training programme)
- Doing something else (please describe)
- Prefer not to say

Q5.6 *In what capacity are you involved as a DBMRI Consumer? Please tick all that apply.

- As a person/patient with experience of a health condition or disease
- As a member of the general public
- As someone with a strong interest in science or health and medical research
- As a carer of someone with a health condition/disease
- Other (please describe)

|  |
| --- |

Q5.7 *Where you born in Australia?

- Yes
- No
- Prefer not to say

Q5.8 *If no, how many years ago did you first arrive in Australia (to live for one year or more)?

- 0 - 5 years
- >5 - 10 years
- >10 - 20 years
- 20+ years ago
- Prefer not to say

Q5.9 *With which broad grouping/s of cultural backgrounds (ie: ethnicity and ancestry) do you identify? Please select all that apply.

- Aboriginal
- Torres Strait Islander
- Anglo-Celtic - English, Scottish, Welsh, Irish
- European - includes all European backgrounds other than Anglo-Celtic eg: German, French, Dutch Italian, Greek, Polish.........
- South-East Asian - eg: Vietnamese, Malaysian....
- North-East Asian - eg: Chinese, Japanese, Korean
- Southern and Central Asian - eg: Indian, Sri-Lankan, Afghani
- Latin American - eg: Mexican, Colombian
- Middle Eastern and North African - eg: Egyptian, Turkish
- Sub-Saharan African - eg: Nigerian, Zimbabwean
- Oceanic and Pacific Islander - eg: Maori, Tongan
- Prefer not to say
- Other, please describe……

Q5.10 *Gender: how do you identify?

- Woman/she/her
- Non-binary/them/their
- Man/he/him
- Prefer to self describe
- Prefer not to say

Q6.1 *On a scale from 1-10, how likely are you to recommend joining the DBMRI as a Consumer to a friend or colleague where 1 is *Not at all likely to recommend* and 10 is *Highly likely to recommend*? (Select a single response from 1 to 10)

Q6.2 **Final Question**: Please share any further thoughts, experiences, suggestions or ideas regarding the DBMRI Consumer Program or Consumer involvement in medical/health research. Open: no limit.
