# Supplementary material for: Patient and public involvement in preclinical and medical research: Evaluation of an established programme in a Discovery‐Based Medical Research Institute
Source: Health Expect. 2024 Jan 19;27(1):e13968. doi: 10.1111/hex.13968 (PMC10797251; doi:10.1111/hex.13968)
Supplement: Supplementary file 2 — Figurementary Figure 2. Online survey for research. [file HEX-27-e13968-s001.docx]

Consumer Program Evaluation - Researcher

Q1.1 Background study information and key elements of consent:

- I consent, begin the study
- I do not consent, I do not wish to participate

*Becoming a Researcher working with a Consumer; please consider the experience of joining the DBMRI program*

Q2.1 *How did your involvement with a Consumer come about? (eg: How did you first become aware of this opportunity at the DBMRI?) Open response: no limit.

Q2.2 *Why did you start working with a Consumer? Open response: no limit.

Q2.3 *In your opinion, what is the purpose and role of consumer involvement in research? Open response: no limit.

Q2.4 *In which DBMRI Research Theme area do you mainly work? Multiple-choice – 5 themes.

Q2.5 *How well prepared did you feel for engaging with a Consumer when you commenced working together, where 1 is *Not at all prepared* and 10 is *Ideally prepared*?

(Select from 1 – 10)

Q2.6 Please comment on your rating.........(eg: what does or could contribute to being ideally prepared?) Optional response: Open: no limit.

Q2.7/2.8 *Please reflect on your experiences and respond to each of the statements about the DBMRI Consumer Program.

|  | Strongly agree (1) | Agree (2) | Neither agree nor disagree (3) | Disagree (4) | Strongly disagree (5) | Not Applicable (6) |
| --- | --- | --- | --- | --- | --- | --- |
| The initial introduction to working with a consumer made my role clear |  |  |  |  |  |  |
| I understand what is expected of me as a researcher working with a Consumer |  |  |  |  |  |  |
| I can easily access the Coordinator of the DBMRI Consumer Program |  |  |  |  |  |  |
| I can access adequate support through the DBMRI for my role as a researcher working with a Consumer |  |  |  |  |  |  |
| My Consumer(s) is (are) an integral part of my research project/ program |  |  |  |  |  |  |

| My manager/ supervisor is supportive of my involvement with my Consumer(s) |  |  |  |  |  |  |
| --- | --- | --- | --- | --- | --- | --- |
| I feel my Consumer(s) was(were) well prepared to take up the role |  |  |  |  |  |  |
| I have a direct say in when I meet/work with my Consumer(s) |  |  |  |  |  |  |
| The times that we meet/work together are convenient for me. |  |  |  |  |  |  |
| Working with my Consumer takes up too much of my time. |  |  |  |  |  |  |

Q2.9 Please share any feedback you have about your experiences of the coordination and organisation aspects of the Consumer Program. Open: no limit

Q2.10 *What was it like meeting your Consumer for the first time? How did you feel? Open: no limit

*How do you work with your Consumer?*

Q3.1 *In what ways does the Consumer contribute to your research, team and/or lab?

|  | Often | Sometimes | Rarely | Never |
| --- | --- | --- | --- | --- |
| Grant application assistance |  |  |  |  |
| Giving feedback to the me/my team about presentations and sharing research results |  |  |  |  |
| Diary/record keeping regarding the partnership |  |  |  |  |
| Mentoring me/my team |  |  |  |  |
| Talking about DBMRI research as a Consumer representative (eg: to community organisations, conferences) |  |  |  |  |
| Involved in writing papers and publications regarding the research |  |  |  |  |
| Teamwork/team building support |  |  |  |  |
| Fundraising support to my research/team |  |  |  |  |
| Networking and connection building |  |  |  |  |
| Personal support to me/my team |  |  |  |  |
| Discussing future research, research questions or research planning |  |  |  |  |
| Other role: If yes, please describe & select how often. If no, select "never". |  |  |  |  |

Please select an answer for each way of working, *including* "Other role". 
Please select "never" if this way of working does not apply.

Q3.2 *Who usually organises activities and meetings with your Consumer(s)?

- The DBMRI Consumer Program Coordinator
- I do
- My Consumer(s) does(do)
- Other, please describe__________________________________________________

Q3.3 *How do you usually interact and work with your Consumer(s)?  Please consider each way of working *including* "Other".  For each way of working, please select Yes/No.

|  | Yes - regularly | Yes - occasionally | No |
| --- | --- | --- | --- |
| Meetings (face-to-face) |  |  |  |
| Meetings via Zoom/Virtually |  |  |  |
| Phone calls |  |  |  |
| Emails |  |  |  |
| Sharing documents via electronic platforms (eg: Teams or Dropbox) |  |  |  |
| Meeting with others outside the DBMRI regarding the research (eg: community groups, funding bodies) |  |  |  |
| Other; If yes, please describe |  |  |  |

Q3.4 *In your experience, which approach or combination of approaches to working together are best and why? Open: no limit

Q3.5 **Including* ***all*** *the different ways of working* together, please select the option that best reflects approximately how often you are in contact with your Consumer(s).

- Multiple times per week
- About weekly
- About fortnightly
- About monthly
- About every 6 - 8 weeks
- About quarterly (approx 4 times/year)
- About twice a year
- Only when there is a grant application to write. Please state approximately how often this occurs in a year.

Q3.6/3.7 *What is the experience like of being a Researcher engaged with Consumers at the DBMRI? Please respond to all statements.

|  | Strongly agree | Agree | Neither agree nor disagree | Disagree | Strongly disagree |
| --- | --- | --- | --- | --- | --- |
| I have enough opportunities to engage with my Consumer(s) about research |  |  |  |  |  |
| I listen to my Consumer's opinion |  |  |  |  |  |
| I sometimes struggle to explain the science and technicalities of my research to my Consumer(s) |  |  |  |  |  |
| I respect my Consumer's opinion |  |  |  |  |  |
| Consumers are a valued member of the team |  |  |  |  |  |
| I feel comfortable working with my Consumer(s) |  |  |  |  |  |
| In my experience, Consumer involvement in research at the DBMRI is more of a "tick box" exercise |  |  |  |  |  |

| The Consumer(s) I work with sometimes struggle to understand my perspective |  |  |  |  |  |
| --- | --- | --- | --- | --- | --- |
| I am able to share my experience and knowledge with my Consumer(s) |  |  |  |  |  |
| I sometimes struggle to understand my Consumer's perspective |  |  |  |  |  |
| I feel that the expectations are too high on me as a Researcher working with a Consumer |  |  |  |  |  |
| In my experience, Consumer involvement in research at the DBMRI is really believed in (11) |  |  |  |  |  |

Q3.8 *In your opinion, does consumer involvement bring added value to DBMRI research?

- Yes
- Maybe
- No

Q3.9 Please comment on your answer about the added value of consumer involvement to DBMRI research (Why/why not......what underpins your opinion?)  Open: no limit

Q3.10 *Do you think that the *researcher(s)* you work with share your view about the added value of consumer involvement to DBMRI Research?

- Definitely yes
- Probably yes
- Might or might not
- Probably not
- Definitely not

Q3.11 *Please comment on your answer............Open: no limit

Q3.12 *Working with my Consumer(s) has altered my practice when writing proposals/applications.

- Yes (please comment on how)
- No (please comment)

Q3.13 *How do you judge whether consumer involvement has made a difference to a study or research program you are involved in? Open: no limit

Q3.14 Please give any examples of how an application, project, program or other aspect of DBMRI research has changed because of consumer involvement..........Open: no limit

Q3.15 What more, if anything, could Consumers do to contribute to DBMRI research? Open: no limit

Q3.16 *What do you think are the downsides of consumer involvement in research? Open: no limit

Q3.17 Please describe anything that has come from the partnership between you and your Consumer(s) that you were not expecting or that has surprised you? Open: no limit.

Q3.18 *Has anything gone wrong or had a negative impact during your involvement in the Consumer Program at the DBMRI?

- Nothing I can think of
- Yes, Please describe……..Open: no limit.

Q3.19 What, if anything, have you learned from the experience of working with a Consumer at the DBMRI? Open: no limit.

Q3.20 *What do you get from the experience of working with a Consumer at the DBMRI? Open: no limit.

Q3.21 *What would you change or improve about the DBMRI Consumer Program? Open: no limit.

*Advice and future planning*

Q4.1 *What do you think are the best ways to enable consumer involvement in research to work well?  What needs to be in place? Open: no limit.

Q4.2 *What do you think are the challenges or barriers to consumer involvement in research working well?  Open: no limit.

Q4.3 *Please describe how COVID-19 has impacted your engagement with your Consumer(s). Open: no limit.

*About You*

Q5.1 *At the start of 2021, for approximately how long had you been involved with a Consumer at the DBMRI? *Please answer in months* eg: 1 year = 12; 2.5 years = 30; 4 years = 48

Q5.2 *What is your age?

- < 20
- 20 - 29
- 30 - 39
- 40 - 49
- 50 - 59
- 60 - 69
- 70 - 79
- 80 +

Q5.3 *What is the highest level of education you have completed or the highest degree you have received?

- High School (up to Year 11)
- High school graduate (Year 12, HSC/VCE or equivalent)
- VET qualification
- Bachelor's degree
- Post Graduate Diploma
- Master's degree
- Professional degree (JD, MD)
- Doctoral degree/PhD
- Other, please specify

Q5.4 *Are you .......

- A PhD student?
- An early career/emerging researcher (eg: within 10 years post PhD or equivalent)?
- A mid-career researcher (10 - 20 years post PhD or equivalent)?
- An established/senior researcher (20+ years post PhD or equivalent)?

Q5.5 *Where you born in Australia?

- Yes
- No (automatically moved to Q5.6)
- Prefer not to say

|  |
| --- |

5.6 *How many years ago did you first arrive in Australia (to live for one year or more)?

- 0 - 5 years
- >5 - 10 years
- >10 - 20 years
- 20+ years ago
- Prefer not to say

Q5.7 *With which broad grouping/s of cultural backgrounds (ie: ethnicity and ancestry) do you identify? Please select all that apply.

- Aboriginal
- Torres Strait Islander
- Anglo-Celtic - English, Scottish, Welsh, Irish
- European - includes all European backgrounds other than Anglo-Celtic eg: German, French, Dutch Italian, Greek, Polish….
- South-East Asian - eg: Vietnamese, Malaysian....
- North-East Asian - eg: Chinese, Japanese, Korean
- Southern and Central Asian - eg: Indian, Sri-Lankan, Afghani
- Latin American - eg: Mexican, Colombian
- Middle Eastern and North African - eg: Egyptian, Turkish
- Sub-Saharan African - eg: Nigerian, Zimbabwean
- Oceanic and Pacific Islander - eg: Maori, Tongan
- Prefer not to say
- Other, please describe

Q5.8 *Gender: how do you identify?

- Woman/she/her
- Non-binary/they/their
- Man/he/him
- Prefer to self describe ______________________________
- Prefer not to say (9)

*Final Questions*

Q6.1 *Thank you for your answers so far. You have just two more questions to go. Once you complete these and click "Submit" your survey will register as completed and you will not be able to return to any answers.*
If you wish to check or revise any of your answers, please do so before clicking the "Submit" button. 


**Final Questions**
*On a scale from 1-10, how likely are you to recommend joining the Consumer program to a DBMRI colleague where 1 is *Not at all likely to recommend* and 10 is *Highly likely to recommend*? (Select one answer from 1 -> 10)

Q6.2 **Final Survey Question**: Please share any further thoughts, experiences, suggestions or ideas you have regarding the DBMRI Consumer Program or consumer involvement in medical/health research. Open: no limit.
