# Supplementary material for: Patient and public involvement in preclinical and medical research: Evaluation of an established programme in a Discovery‐Based Medical Research Institute
Source: Health Expect. 2024 Jan 19;27(1):e13968. doi: 10.1111/hex.13968 (PMC10797251; doi:10.1111/hex.13968)
Supplement: Supplementary file 3 — Figurementary Figure 3. Interview Guide. [file HEX-27-e13968-s002.docx]

**Supplementary Figure 3**

**Interview Guide – Consumer Involvement Program Study**

Indicative questions developed for Consumers/Researchers

*Preamble conversation – talk about project/context*

*(Opportunity to ask questions/clarify with interviewer)*

- Explore views on purpose of consumer involvement and role of consumers in medical research broadly (not just this program)

*Explore role in program*

- How did you first become aware of the Program?

(awareness/engagement)

Tell me about how you came to be involved in the consumer program in the first place?

- What prompted/ motivated you? (explore the decision…… any other Consumer involvement experiences elsewhere?)
- How long involved?
- Tell me about your role, and what is expected of you in the program
- What was your experience of starting out in the program. ……(experience of induction/orientation/initial training )
- How well did the training and/or support from the organisation prepare/support you for your role?
- What could be done differently in terms of preparation/support? (for you – for your research partners?)

*Explore program experiences*

- What’s your experience of the coordination and organisation of the program?

Eg: communication, appointment times

- What would you say are the key steps needed to build a working relationship with a researcher/consumer partner?
- How often do you work with you partner(s)? Explore time and engagement.

(a) What do you do? Describe how you work together

(b) Has this evolved over time? If so, how?

- What is the relationship like with your research/consumer partner(s)…….

(For example; communication, respect, valuing expertise, who “chases” who inclusiveness, partnership?)

- How comfortable do you feel working with your research/consumer partner(s)? (Explore if possible)
- In your view, does the partnership have an influence on the research? (Explore to understand reasons/explanation….)
- What (if anything), comes from the partnership (s)? What has surprised you?
- Has anything gone wrong, or had a negative impact for you during your involvement in the program? Tell me a bit about that…….
- What have you learned from the experience of being part of the program? (positives/neutral/negatives……..). What personally do you get from being part of the program?
- Tell me about the best thing(s) about the program? ……and the most challenging thing (s)?
- Would you recommend joining the program to others? Tell me a bit more about your answer (why/why not…..)

*Program feedback*

- What do you think are the qualities and attributes of the ideal consumer/researcher partner?
- What things make it easy to be involved in the program (the enablers)?
- What things make it difficult to be involved in the program (the barriers)?
- What advice would you give to people new to the program…..

*Consumers/researchers/ partnerships…..*

- What advice if any, would you give to organization about the program? What would you change about the program?
- What advice would you give to other organisations setting up a similar program?
- (If not already mentioned) – ask How has COVID-19 impacted your participation in the program?

*Explore if anything else the person would like to share*………what further thoughts, experiences, suggestions or ideas do you have to share?
